# Skeletal Effects of the Saturated 3-Thia Fatty Acid Tetradecylthioacetic Acid in Rats

**DOI:** 10.1155/2011/436358

**Published:** 2011-12-05

**Authors:** Astrid Kamilla Stunes, Irene Westbroek, Reidar Fossmark, Rolf Kristian Berge, Janne Elin Reseland, Unni Syversen

**Affiliations:** ^1^Department of Cancer Research and Molecular Medicine, Faculty of Medicine, MTFS, Norwegian University of Science and Technology, NTNU, Olav Kyrres gate 9, 7489 Trondheim, Norway; ^2^Departments of Orthopedics and Internal Medicine, Erasmus University, 3000 DR, Rotterdam, The Netherlands; ^3^Department of Gastroenterology and Hepatology, St. Olav's University Hospital, 7006 Trondheim, Norway; ^4^Department of Medicine, University of Bergen, 5020 Bergen, Norway; ^5^Department of Heart Diseases, Haukeland University Hospital, 5021 Bergen, Norway; ^6^Department of Biomaterials, Institute for Clinical Dentistry, University of Oslo, 0317 Oslo, Norway; ^7^Department of Endocrinology, St. Olav's University Hospital, 7030 Trondheim, Norway

## Abstract

This study explores the skeletal effects of the peroxisome proliferator activated receptor (PPAR)pan agonist tetradecylthioacetic acid (TTA). Rats, without (Study I) and with ovariectomy (OVX) or sham operation (Study II), were given TTA or vehicle daily for 4 months. Bone markers in plasma, whole body and femoral bone mineral density and content (BMD and BMC), and body composition were examined. Histomorphometric and biomechanical analyses (Study I) and biomechanical and *μ*CT analyses (Study II) of the femur were performed. Normal rats fed TTA had higher femoral BMD and increased total and cortical area in femur compared to controls. The ovariectomized groups had decreased BMD and impaired microarchitecture parameters compared to SHAM. However, the TTA OVX group maintained femoral BMC, trabecular thickness in the femoral head, and cortical volume in the femoral metaphysis as SHAM. TTA might increase BMD and exert a light preventive effect on estrogen-related bone loss in rats.

## 1. Introduction

 Fatty acids are natural ligands for peroxisome proliferator activated receptors (PPAR) *α*, *δ*, and *γ*. Tetradecylthioacetic acid (TTA) is a saturated 16 carbon 3-thia synthetic fatty acid, which also acts as a PPARpan agonist.

Studies have shown multifaceted effects of TTA in rodents. It induces mitochondrial fatty acid *β*-oxidation and causes reduced plasma levels of free fatty acids (FFA) and triglycerides (TG) in addition to improved insulin response, anti-inflammatory action and lowered oxidative stress [1–3]. The responses are qualitatively similar to the effects of *n*-3 fatty acids, but TTA seems to have a greater biological potency. A main determinant of the mechanism of action seems to be the non-*β*-oxidizability of TTA due to the sulphur atom in the third position in the carbon chain [4]. 

TTA is also found to attenuate dyslipidemia in male patients with type 2 diabetes mellitus [1]. Many of the effects of TTA in this study point in direction of PPAR*α* as well as PPAR*δ* activation, with a similar pattern of lipid lowering effects as observed in patients with impaired glucose tolerance after treatment with fenofibrate, which is a selective PPAR*α* agonist [1, 5]. Studies in rodents also indicate that TTA acts through mechanisms that at least partly involve PPAR*α* activation [2].

All PPAR subtypes are present in bone cells [6]. We and others have shown that PPAR*γ* agonists have negative skeletal effects in rodents, by decreasing bone mass and deteriorating the architectural structure [7–11]. Increased fracture incidence has also been observed in patients with type 2 diabetes treated with glitazones [12–15]. In contrast, we have demonstrated that administration of fenofibrate has a positive impact on bone, both in normal intact female rats and ovariectomized rats [7, 8]. The PPAR*α* agonist bezafibrate and the PPAR*δ*/*α* agonist linoleic acid administered to rats are also found to increase bone mass and bone formation [16].

Cornish et al. reported that saturated fatty acids inhibit osteoclastogenesis [17]. In accordance with this, endogenous *n*-3 fatty acids have been shown to protect against bone loss by attenuating osteoclastogenesis in ovariectomized mice [18], and high consumption of fish, a rich source of omega-3 fatty acids, protected against bone loss during long-duration spaceflight [19].

The PPARpan agonist TTA has been reported to exhibit multiple positive biological effects; however, the effects of TTA on bone have so far not been explored. Due to the different effects of PPAR*γ* agonists and PPAR*α* agonists on bone metabolism, we aimed to examine the skeletal effect of TTA administration in intact as well as in ovariectomized female rats. Furthermore, we studied the effect of TTA on proliferation and differentiation of bone cells and release of osteoprotegerin (OPG) and secreted receptor activator of nuclear factor *κ*B ligand (sRANKL) from preosteoblasts.

## 2. Methods

TTA was provided by one of the coauthors. Methylcellulose (M7140, Sigma-Aldrich, St. Louis, MO) was used as solvent/vehicle. Female Fischer-344 and Sprague-Dawley rats were purchased from Møllegaard's Breeding Center (Skensved, Denmark).

### 2.1. Design of Studies

The Animal Welfare Committee at the St. Olav's University Hospital in Trondheim approved the studies. All rats were housed solely in wire-top cages with aspen woodchip bedding (B&K Universal Ltd, UK). Room temperature was 24 ± 1.0°C with a relative humidity of 40%–50% and a 12-hour light/dark cycle. The Rat and Mouse Diet (B&K) and tap water were provided *ad libitum*. The *in vivo* studies were performed simultaneously with two previous published studies, and the controls used are the same as in [8] for Study I and in [7] for Study II.

**Study I**

Twenty-four female Fischer rats, eight weeks of age (203 ± 11 g), were randomly assigned to two groups, one group received methylcellulose (CTR, *N* = 12) and the other group methylcellulose with TTA (TTA, *N* = 12) 35 mg/kg body weight, daily for four months by intragastric gavage.

**Study II**

Thirty-three female Sprague-Dawley rats, twelve weeks of age (252 ± 16 g), were randomly assigned to three groups of eleven rats. Ovariectomy is a well-known and widely used model system for osteoporosis in rodents [20, 21] and was performed in this study. One group was sham-operated and given methylcellulose (vehicle) (CTR SHAM), the two other groups were ovariectomized and given methylcellulose (CTR OVX) or TTA (90 mg/kg body weight) (TTA OVX), by daily intragastric gavage for four months. We applied a higher dose of TTA in Study II, in accordance with doses previously used in other *in vivo *studies in rats, without signs of toxicity [22, 23].

For both studies, the rats were weighed at the start and monthly throughout the studies. Before all procedures and sacrifice, the animals were anesthetized with 2.0 mL/kg body weight of a combination of fluanison (2.5 mg/mL), fentanyl (0.05 mg/mL), and midazolam (1.25 mg/mL). Blood was collected by heart puncture during the final anesthesia and stored at −80°C until analyses. Liver weights were registered and femurs from all animals were collected, measured, and stored at −80°C for further analyses.

### 2.2. Dual X-Ray Absorptiometry (DXA) Measurements

Body weight (g), fat mass (g), lean mass (g), bone mineral content (BMC) (g), and whole body and femur bone mineral density (BMD) (g/cm^2^) were measured by DXA in anesthetized animals, using a Hologic QDR 4500A and small animal software. DXA was performed in both Study I and Study II, at the beginning of the studies and after two and four months. The coefficient of variation (CV) was 2.4% for body weight, 2.2% for fat mass, 0.28% for lean mass, 0.54% for whole body BMC, 3.0% for femur BMC, 0.60% for whole body BMD, and 0.71% for femur BMD.

### 2.3. Histomorphometry

Histomorphometry of the left femur was performed in Study I and evaluated by two persons. Transverse sections were cut close to the patellar ridge and also 5.0 mm proximal from it. They were grounded to a thickness of 50 um. The sections close to the patellar ridge were used for calculation of trabecular bone volume (TBV%), and proximal sections were used for calculation of cortical thickness. The sections for calculation of TBV% were stained with Goldner. For calculation of TBV%, a Merz grid at a magnification of 10 × 10 was used. As many squares as possible, usually about 9–11, were calculated. For calculation of cortical thickness the central point of the medullary canal was identified. By means of an eyepiece the diameters were calculated in four directions with a 45-degree angle between each. Medullary, cortical and total cross-sectional areas were calculated from the mean values. All histomorphometric measurements were performed blindly.

### 2.4. Microcomputed Tomography (*μ*CT) Measurements

In Study II, the proximal femurs, including the femoral head and the metaphysis of the dissected femurs, were scanned in a SkyScan 1072 microtomography (SkyScan, Antwerp, Belgium), with a voxel size of 11.89 *μ*m. Scans were processed, and three-dimensional morphometric analyses of the femurs were done using free software of the 3D-Calc Project (http://www.erasmusmc.nl/47460/386156/Downloads?lang =en). Femoral head and metaphysis data sets were analyzed separately. Cortical bone and trabecular bone were separated using in-house developed software. For each cross-section in the 3D data set, a virtual mask of the total bone was created and used to identify the bone marrow regions in the original image. The marrow regions were expanded into a mask of the total marrow cavity using a close operation. Bone inside the total marrow cavity was considered trabecular bone; the remainder was regarded as cortex. Cortical bone volume (Ct.V, *μ*m^3^), cortical thickness (Ct.Th, *μ*m), trabecular bone volume (BV, *μ*m^3^), total bone volume, the region of interest adjacent to the endocortical boundary including the trabecular bone as previously described [24] (TV, *μ*m^3^), trabecular thickness (Tb.Th, *μ*m), trabecular bone volume fraction (BV/TV), connectivity density (CD, 1/mm^3^) [25], and structure model index (SMI, (0–3)) [26] were determined. SMI indicates whether the trabeculae are more rodlike (SMI = 3) or more platelike (SMI = 0), and values between 0 and 3 represent the volume ratio of rods and plates, analyzed as previously described [26].

### 2.5. Biomechanical Testing

In Study I and Study II, the right femurs were thawed in Ringer's solution before mechanical testing of the femoral neck and shaft was performed. The shafts were fractured 18.7 mm from the femoral condyles in three-point cantilever bending as previously described [27]. In short, the proximal femur was fixed in a clamp, the cam of the rotating wheel engaged the femoral condyles, and a fulcrum positioned 18.7 mm anteriorly from the condyles was the third-point of force application. All tests were done at a loading rate of 0.095 radians/second (5.43°/second). The load in the test apparatus, a MTS 858 Mini Bionix Axial/Torsional Test System (MTS Systems Corporation, Minn, USA), was measured with an MTS Test Star TM Sensor Cartridge Force 250 N load cell and registered in MTS Test Star II software. Ultimate bending moment (*M*) was calculated as the ultimate load before failure multiplied by the moment arm by which the load was applied (Newton Meter, Nm). Energy absorption and stiffness were read directly or calculated from the computer recordings as previously described [27].

### 2.6. Plasma Analyses

Osteocalcin in plasma was determined by a Rat-MID osteocalcin enzyme-linked immunosorbent assay kit (Nordic Bioscience Diagnostics A/S, Denmark), according to the manufacturer's protocol. The detection limit was 50 ng/mL, and intra- and interassay variations were 5.0% and 5.5%, respectively. The bone resorption marker CTX in plasma was analyzed by a RatLaps ELISA kit (Nordic Bioscience Diagnostics A/S) according to the instructions from the manufacturer. The detection limit was 3.0 ng/mL, and intra- and interassay variations were 5.6% and 10.5%, respectively.

### 2.7. Cells and Reagents

MC3T3-E1 (mouse preosteoblasts, ATCC) and RAW264.7 (mouse preosteoclasts, ATCC) were maintained in MEM*α* or Dulbecco's Modified Eagle Medium (DMEM), respectively, (Invitrogen) supplemented with 10% fetal calf serum (FCS) (EuroClone, Great Britain), 1 mM sodium pyruvate (Gibco BRL, Life Technologies Ltd, Scotland), 0.1 mg/mL L-glutamine (Gibco), and 10 U/mL penicillin/streptomycin (Gibco).

### 2.8. Proliferation Assay

Proliferation of MC3T3-E1 and RAW 264.7 cells was studied using 5-bromo-2′-deoxyuridine (BrdU) incorporation (Roche Molecular Biochemicals, Mannheim, Germany) as previously described [8].

### 2.9. Osteoprotegerin (OPG), Secreted Receptor Activator of Nuclear Factor *κ*B Ligand (sRANKL) and Total Protein Analyses in Cell Medium

The concentration of OPG and sRANKL in culture media from preosteoblasts (MC3T3-E1cells) exposed to TTA was determined by OPG ELISA (RnD Systems, Minneapolis, Minn, US) and a sRANKL ELISA (Biomedica, Vienna, Austria) as previously described [8, 28], respectively. The amount of OPG and sRANKL was related to the amount of total protein in each sample, which was measured by a Bradford assay [29] using a dye concentrate (Bio-Rad, Hercules, Calif, US).

### 2.10. Bone Marrow Cell Preparation

Bone marrow cells were obtained from femurs and tibiae from three intact female Sprague Dawley rats (251 ± 4.5 g) by centrifugation as described by Dobson et al. [30]. Briefly, tibiae and femurs were removed and all soft tissue was removed. The proximal ends were cut off, and the bones briefly centrifuged (1000 g, 10 s). The bone marrow pellets from each animal were pooled and resuspended in culture medium and passed through a 21-gauge needle to achieve a single-cell suspension.

### 2.11. Mineralizing Fibroblast-Colony-Forming Unit Cultures Assays (mCFU-f)

For *in vitro* studies: Dulbecco's Modified Eagle Medium (DMEM) was supplemented with penicillin/streptomycin (10 U/mL), L-glutamine (0.1 mg/mL), sodium pyruvate (1.0 mM), (all from Gibco BRL Invitrogen, Paisley, UK). MCFU-f cultures assays were performed essentially as previously described by Scutt et al. [31]. Nucleated bone marrow cells from three female rats were seeded in DMEM/10% fetal calf serum (FCS) (EuroClone, Devon, UK) with 10 nM dexamethasone (Sigma, Oslo, Norway), 50 *μ*g/mL ascorbic acid (Sigma), and 2.0 mM *β*-glycerophosphate, in 6 well-plates (1.0 × 10^6^ cells/well). Cells were treated with vehicle (medium) and TTA (1.0 and 10 *μ*M). The media were changed after 5 days and thereafter every second day for 21 days. Cultures were terminated by washing with phosphate-buffered saline (PBS) and fixed by adding cold 100% ethanol. Wells were stained for alkaline phosphatase (ALP) by addition of 0.5 mg/mL napthol AS-BI phosphate (Sigma) and 1.0 mg/mL fast red B (Sigma) in Tris buffer (pH 7.5) for 30 minutes, washed with distilled water, photographed with a digital camera, and destained with 100% ethanol over night. Calcium-positive colonies were stained with 1.0 mg/mL alizarin red (Sigma) in distilled water adjusted to pH 5.5 with NH_3_ for 30 minutes, washed with distilled water, photographed, and destained with 5% perchloric acid for 5 minutes. Collagen-positive colonies were stained with 1.0 mg/mL sirius red (Sigma) in statured picric acid for 18 hours, washed with distilled water, and photographed. Picture processing was performed in Adobe Photoshop software (Adobe, San Jose, Calif, USA) and quantification of colonies was performed using the ImageQuant Software (Amersham Biosciences, Piscataway, NJ, USA).

### 2.12. Statistical Analyses

All measurements were performed in duplicates or triplets. Data are expressed as mean ± SD or mean ± SEM, as indicated in figures and tables. All data were tested for normality with the D'Agostino-Pearson omnibus normality test. Normally distributed parameters were tested with two-tailed unpaired student's *t*-test, or one-way ANOVA with Bonferroni's post test, while parameters without normal distribution were tested with Mann-Whitney's two-tailed test or Kruskal-Wallis with Dunn's post test. *P*-values below 0.05 were considered significant. In Study II, all statistical analyses were performed with mutual comparison between all three groups.

## 3. Results

### 3.1. Study I

#### 3.1.1. General Observations and Body Composition

No differences in the well-being of the animals were observed, and none of the animals died or became ill during the study. There were no differences in body weight, lean or fat mass at baseline or at the end of the study (Table 1). The liver weights were significantly higher in rats given TTA compared to controls (6.92 ± 0.47 g versus 6.32 ± 0.47 g, *P* = 0.005), as described for PPAR*α* agonists previously [7]. We did not detect any liver pathology by gross visual inspection; however, livers were not further examined, as this was not within the scope of this study. Femur lengths were similar in the two groups at the end of the study (data not shown).

#### 3.1.2. Whole Body and Femur BMC and BMD

There were no differences between the two groups at the beginning of the study in whole body BMD or whole body and femur BMC between the two groups (Table 1), while the femoral BMD was significantly higher in rats given TTA for four months, compared to controls (0.256 ± 0.011 g versus 0.245 ± 0.009 g, *P* = 0.012), (Table 1).

#### 3.1.3. Histomorphometry and Biomechanical Properties

Rats fed TTA had significantly higher total area (*P* = 0.015) and cortical area (*P* = 0.02) than control rats in the distal femur (Table 2). There were no significant differences in total medullary area or trabecular bone volume % in the distal femur (Table 2). There were no significant differences in mechanical parameters (ultimate bending moment, stiffness, and energy absorption) of the femoral neck and shaft (Table 3).

#### 3.1.4. Plasma Measurements

There were no significant differences in plasma levels of osteocalcin (CTR: 371 ± 96 ng/mL versus TTA:  386 ± 83 ng/mL) or CTX (CTR: 55.4 ± 42.8 ng/mL versus TTA: 60.7 ± 38.2 ng/mL).

### 3.2. Study II

#### 3.2.1. General Observations and Body Composition

There were no significant differences between the groups at baseline, regarding body weight, fat or lean mass. The TTA OVX group had significantly higher liver weights than the two other groups (TTA OVX: 9.31 ± 0.67 g versus CTR SHAM: 7.57 ± 1.04 g, *P* = 0.0004 and versus CTR OVX: 7.67 ± 0.96 g, *P* = 0.0005). No differences in body composition between the two ovariectomized groups were observed at the end of the study (Table 4), not even in lean mass when corrected for the increased liver weights in the TTA OVX group (data not shown). As expected, the ovariectomized groups had significantly higher body weight, fat and lean mass than CTR SHAM after both two and four months (Table 4). The femoral lengths were similar in all groups at the end of the study (CTR SHAM: 36.4 ± 0.80 mm, CTR OVX: 37.2 ± 0.89 mm and TTA OVX: 37.3 ± 0.89 mm).

#### 3.2.2. Whole Body and Femur BMC and BMD

BMC or BMD were equal between the three groups at the beginning of the study. The TTA OVX group maintained femoral BMC at SHAM levels, in contrast to the OVX controls which had significantly decreased femoral BMC compared to CTR SHAM after four months of treatment (Table 4). No significant differences were observed in total body BMC between the three groups, while both the CTR OVX and the TTA OVX groups had significantly decreased whole body and femoral BMD compared to the CTR SHAM group (Table 2).

#### 3.2.3. Bone Architecture

*μ*CT measurements revealed that ovariectomized rats fed TTA maintained the trabecular thickness (Tb.Th) in the femoral head and the cortical volume (Ct.V) in the femoral metaphysis, at the same levels as the CTR SHAM group. This is in contrast to ovariectomized controls, which had significantly lower Tb.Th and Ct.V compared to the CTR SHAM group (Tables 5 and 6).

The other parameters measured by *μ*CT mainly showed that both ovariectomized groups exhibited impaired bone architecture compared to CTR SHAM (Tables 5 and 6); however, there were no significant differences between the two ovariectomized groups. No significant differences in connectivity density (CD) were registered between any of the groups (Tables 5 and 6).

#### 3.2.4. Biomechanical Properties

Both the CTR OVX and the TTA OVX groups had a significant decrease in bending moment in the femoral neck compared to CTR SHAM (Table 7). However, unlike the CTR OVX group, the energy absorption in the femoral neck in the TTA OVX group was unchanged compared to CTR SHAM (Table 7).

#### 3.2.5. Plasma Measurements

Plasma osteocalcin was significantly elevated in both the ovariectomized groups compared to the CTR SHAM group, while there was no significant difference between the CTR OVX and TTA OVX groups (CTR SHAM: 367 ± 1.04 ng/mL versus TTA OVX: 459 ± 88.4 ng/mL, *P* = 0.012 and versus CTR OVX: 465 ± 139 ng/mL, *P* = 0.05). There were no differences in plasma CTX between the groups (CTR SHAM: 21.7 ± 14.5 ng/mL, CTR OVX: 25.9 ± 2.8, TTA OVX: 21.7 ± 13.5 ng/mL).

### 3.3. In Vitro Studies

#### 3.3.1. Effect of TTA on Bone Progenitors Proliferation

**Figure 1:**
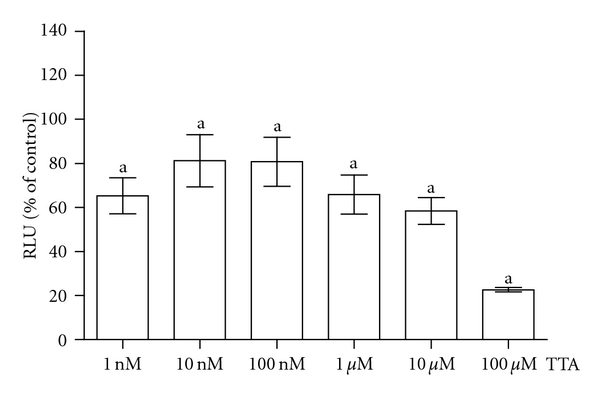
The effect of TTA (1 nm–100 *μ*M) on RAW 264.7 preosteoclast cell proliferation. Data are in mean ± SD and presented as relative light units (RLU) in % of control (untreated cells at same time point), ^a^ *P* < 0.05 when compared to control.

TTA inhibited proliferation of RAW 264.7 preosteoclasts in a dose-dependent manner (20–40%) (Figure 1), but had no effect on MC3T3-E1 preosteoblast proliferation at the doses used (1.0 nM–10 *μ*M) (data not shown). 

#### 3.3.2. Effect of TTA on OPG and sRANKL Release from MC3T3-E1 Preosteoblasts

**Figure 2:**
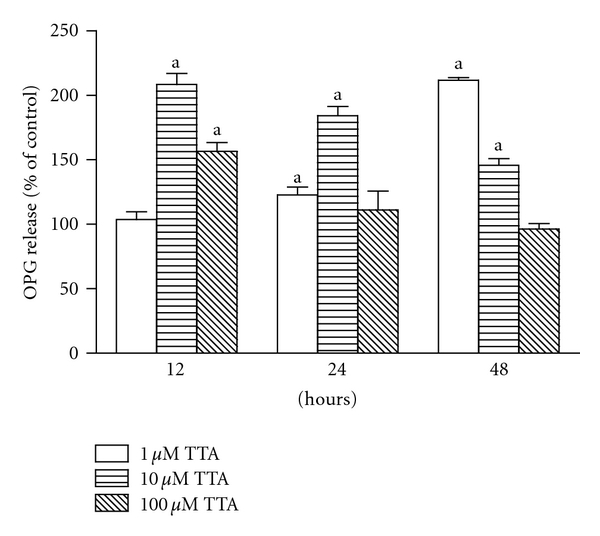
The effect of TTA (1.0, 10 and 100 *μ*M) on osteoprotegerin (OPG) release to the cell medium from MC3T3-E1 preosteoblast cells after 12, 24, and 48 h of stimulation. Data are in mean ± SD and presented as OPG related to the amount of total protein and as % of control (untreated cells at same time point), ^a^ *P* < 0.05 when compared to control.

TTA increased OPG release from MC3T3-E1 preosteoblasts (1.0 and 10 *μ*M, after 12, 24, and 48 h) (Figure 2), but had no significant effects on sRANKL release (data not shown).

#### 3.3.3. Differentiation of Rat Bone Marrow Cells

TTA (1.0 and 10 *μ*M) slightly increased the number of ALP-positive colonies in rat bone marrow cells examined with the mCFU-f assay, however, this did not reach significance (TTA 1.0 *μ*M: 120 ± 4.2  in % of CTR, TTA 10 *μ*M: 125 ± 5.6 in % of CTR). There was no difference in the number of calcium and collagen-positive colonies between rat bone marrow cells treated with TTA (1.0 and 10 *μ*M) and control cells (data not shown).

## 4. Discussion

In the present study we provide the first evidence for positive skeletal effects of TTA. In intact female rats, we observed an increase in femoral BMD compared to controls. Total and cortical areas of femur were also higher; this was, however, not reflected in superior mechanical properties. Ovariectomized rats receiving TTA maintained femoral BMC at the SHAM level, while femoral BMD as well as whole body BMC and BMD, like CTR OVX, decreased compared to sham controls. A few microarchitecture parameters were preserved at SHAM levels in ovariectomized rats fed TTA; trabecular thickness in the femoral head and cortical volume in the femoral metaphysic were maintained at SHAM level. This indicates some protection of TTA against the bone loss induced by estrogen deficiency. Endocortical volume was, however, significantly increased both in the femoral head and metaphysis compared to SHAM. Our data are in accordance with previous studies showing a preventive effect of omega-3 fatty acids on bone loss [17, 19].

The ovariectomized control rats in Study II had both significantly lower gain in femoral BMC and BMD and reduced biomechanical strength parameters compared to sham-operated controls. The relatively young age of the rats may explain why the differences found between the SHAM and OVX controls were less pronounced than expected. According to Kharode et al. [32], rats from 2–15 months are usually applied in the OVX model, and while use of older animals is attractive due to steady bone turnover rate, the use of young adult rats can also provide consistent, reproducible, and interpretable results. Ovariectomy of skeletally immature rats results in achievement of a lower peak bone mass (total bone mass present at the end of the skeletal maturation) which for rats is considered to occur between 47–61 weeks of age [33].

There was no difference in osteocalcin levels in Study I, while in Study II, circulating osteocalcin was increased in both the OVX groups. It is difficult to differentiate if the elevated level of plasma osteocalcin level in the TTA group is due to increased bone formation or increased bone turnover associated with ovariectomy. We have previously shown that rats that received the PPAR*α* agonist fenofibrate had increased osteocalcin levels, both in intact and ovariectomized rats, and that fenofibrate stimulates MC3T3-E1 osteoblast differentiation *in vitro* [7, 8]. We were not able to demonstrate any significant effect of TTA on osteoblast differentiation of rat bone marrow cells in our study; however, a tendency towards increased ALP-positive colonies was observed. Still et al. demonstrated that the observed increase in bone mass induced by the PPAR*α*/*δ* agonists linoleic acid and bezafibrate was a result of increased bone formation in mice [16].

The levels of the plasma bone resorption marker CTX was similar in all groups in both Study I and II. This could be due to fact that the rats were not fasted. We find that TTA stimulates the release of OPG from preosteoblasts, without affecting the release of sRANKL, which implies an inhibitory effect on bone resorption. We also demonstrate that TTA inhibits proliferation of RAW 264.7 preosteoclasts in a dose-dependent manner, which indicates that TTA might exert an inhibitory effect on osteoclast precursor recruitment. In spite of the *in vitro* findings, we observed no difference in total volume between the TTA OVX group and the CTR OVX, indicating that TTA was not able to prevent the increased endosteal bone resorption caused by estrogen deficiency. Medullary area in intact rats was similar in both the TTA and the control groups.

Previously, it has been demonstrated that TTA acts as a PPARpan agonist *in vitro* [34]. However, the potency of TTA-mediated PPAR transactivation and the degree of TTA-induced activation of the individual PPAR subtypes seem to be a subject of cell type specific variation [34]. The affinity for different PPARs in bone cells has so far not been investigated. TTA is found to activate all PPAR subtypes in the ranking order PPAR*α* > PPAR*δ* > PPAR*γ* in rodent cells [35]. The PPAR*α* agonist fenofibrate increases BMD in intact female rats and maintains BMD, microarchitecture, and mechanical strength in ovariectomized rats, in contrast to the negative effects of the PPAR*γ* agonist pioglitazone [7].

The skeletal effects observed in the present study are less pronounced than what we found for fenofibrate [7]. This may be due to a lower potency for PPAR*α*, combined with a possible coactivation of PPAR*γ*. Due to the negative effects of PPAR*γ* agonists on bone, it is possible that TTA, as a PPARpan agonist, might cause a harmful skeletal effect at very high doses. This was, however, not observed in our study. The effect of PPAR*δ* activation on bone is so far not elucidated.

Recently, a new class of free fatty acids receptors has been identified [36, 37]. G-protein receptor (GRP), 41 and GRP43 are activated by short-chain FFAs, whereas GPR40 and GPR120 can be activated by medium- and long-chain FFAs [36, 37]. Cornish et al. have described the expression of GRP120 in osteoblasts as well as osteoclasts, while GRP40, 41, and 43 was expressed in osteoclasts only [17]. TTA may exert PPAR independent effects [35], possibly through activation of GRP receptors in bone cells.

Recently, in a double-blinded, placebo-controlled study, we assessed the metabolic effects of systemic TTA in patients with mild to moderate psoriasis, 1.0 g TTA for 28 days [38]. The most important findings were that TTA reduced the plasma lipids (cholesterol, triglycerides, and total fatty acid) and showed anti-inflammatory effects as it lowered the plasma tumor necrosis factor-*α* and vascular cell adhesion molecule-1. No significant adverse effects or other complications were reported throughout the study. All safety parameters remained within the normal limits both for males and females in the placebo and TTA group [38].

In conclusion, we show that the modified fatty acid TTA seems to have some positive skeletal effects, by increasing femoral BMD in intact female rats and partly preventing bone loss and impairment of microarchitecture in ovariectomized rats. Since TTA might be used in the treatment of metabolic syndrome, it is of importance to show that TTA has no harmful effects on bone like PPAR*γ* agonists as glitazones.

## Figures and Tables

**Table 1:** Body weight, fat and lean mass, femoral and whole body bone mineral content (BMC), and bone mineral density (BMD) in rats at baseline and after four months of treatment with TTA (Study I).

	Months	CTR (controls given vehicle) (*N* = 12)	TTA (given TTA) (*N* = 12)
Body weight (g)	0	215 ± 14.0	220 ± 7.43
	4	235 ± 15.9	242 ± 10.8
Fat mass (g)	0	43.6 ± 10.9	48.8 ± 7.98
	4	46.8 ± 13.7	48.2 ± 14.5
Lean mass (g)	0	165 ± 8.44	160 ± 8.44
	4	183 ± 7.73	186 ± 9.36
Whole body BMC (g)	0	6.38 ± 0.35	6.39 ± 0.35
	4	7.24 ± 0.36	7.31 ± 0.37
Femoral BMC (g)	0	0.32 ± 0.029	0.33 ± 0.014
	4	0.72 ± 0.036	0.73 ± 0.037
Whole body BMD (g/cm^2^)	0	0.142 ± 0.007	0.144 ± 0.010
	4	0.159 ± 0.008	0.154 ± 0.005
Femoral BMD (g/cm^2^)	0	0.233 + 0.010	0.235 ± 0.006
	4	0.245 ± 0.009	0.256 ± 0.011^a^

Data are presented as mean ± SD, ^a^ *P* < 0.05  significantly different compared to control.

**Table 2:** Histomorphometric analysis of trabecular bone volume, total, cortical, and medullary area in distal femur after four months of treatment with TTA (Study I).

Group	CTR (controls given vehicle) (*N* = 12)	TTA (given TTA) (*N* = 12)
Trabecular bone volume (%)	22.2 ± 5.95	22.1 ± 4.70
Total area (mm^2^)	6.26 ± 0.32	6.65 ± 0.32^a^
Cortical area (mm^2^)	3.99 ± 0.29	4.27 ± 0.24^a^
Medullary area (mm^2^)	2.35 ± 0.25	2.37 ± 0.15

Data are presented as mean ± SD, ^a^ *P* < 0.05 significantly different compared to control.

**Table 3:** Mechanical properties of femoral neck and shaft after four months of treatment with TTA (Study I).

Group	CTR (controls given vehicle) (*N* = 12)	TTA (given TTA) (*N* = 12)
Ultimate bending moment (Nm)		
Femoral neck	50.8 ± 5.17	49.3 ± 8.86
Femoral shaft	48.3 ± 3.41	45.3 ± 4.01
Energy absorption (J × 10^−2^)		
Femoral neck	119 ± 12.0	119 ± 16.0
Femoral shaft	112 ± 8.00	106 ± 9.00
Bending stiffness (Nm/° × 10^−3^)		
Femoral neck	0.78 ± 0.10	0.75 ± 0.11
Femoral shaft	1.05 ± 0.18	1.04 ± 0.14

**Table 4:** Body weight, fat and lean mass, femoral and whole body bone mineral content (BMC), and bone mineral density (BMD) in mean values ± SEM in sham controls, ovariectomized controls, and ovariectomized rats fed TTA, at baseline, and after two and four months (Study II).

	Months	SHAM (sham-operated controls) (*N* = 10)	OVX (ovariectomized controls) (*N* = 10)	TTA OVX (ovariectomized, given TTA) (*N* = 10)
Body weight (g)	0	264.1 ± 5.6	277.3 ± 4.5	273.7 ± 11.7
	2	280.6 ± 6.0	339.2 ± 5.7^aaa^	338.2 ± 16.2^aaa^
	4	288.9 ± 6.0	351.8 ± 7.1^aaa^	352.5 ± 17.8^aaa^

Fat mass (g)	0	21.6 ± 2.6	18.2 ± 1.9	20.1 ± 4.2
	2	27.3 ± 1.6	38.8 ± 3.0^aaa^	37.2 ± 7.2^aaa^
	4	28.3 ± 2.1	55.8 ± 4.9^aaa^	51.1 ± 8.4^aaa^

Lean mass (g)	0	233.9 ± 3.5	250.7 ± 3.8	248.4 ± 14.4
	2	244.0 ± 5.5	290.1 ± 5.6^aaa^	291.0 ± 20.0^aaa^
	4	251.1 ± 4.9	285.7 ± 5.9^aaa^	291.0 ± 14.9^aaa^

Whole body BMC (g)	0	8.51 ± 0.16	8.41 ± 0.16	8.69 ± 0.52
	2	9.34 ± 0.16	9.81 ± 0.20	10.07 ± 0.48
	4	9.56 ± 0.18	10.33 ± 0.19	10.32 ± 0.48

Femur BMC (g)	0	0.418 ± 0.005	0.409 ± 0.010	0.430 ± 0.030
	2	0.463 ± 0.011	0.444 ± 0.008	0.464 ± 0.025
	4	0.489 ± 0.011	0.456 ± 0.007^a^	0.471 ± 0.025

Whole body BMD (g/cm^2^)	0	0.173 ± 0.001	0.171 ± 0.001	0.175 ± 0.005
	2	0.185 ± 0.002	0.179 ± 0.002	0.181 ± 0.005
	4	0.184 ± 0.001	0.178 ± 0.002^aaa^	0.178 ± 0.004^a^

Femur BMD (g/cm^2^)	0	0.305 ± 0.006	0.292 ± 0.004	0.302 ± 0.017
	2	0.312 ± 0.004	0.293 ± 0.002	0.299 ± 0.010
	4	0.319 ± 0.004	0.294 ± 0.003^aaa^	0.295 ± 0.009^aa^

^a,  aa,  aaa^ *P* < 0.05, *P* < 0.01, *P* < 0.001 when compared to CTR SHAM group.

**Table 5:** Mean values ± SD of bone architecture parameters in the femoral head, determined by means of *μ*CT scanning analyses in sham controls, ovariectomized controls, and ovariectomized rats fed TTA for four months (Study II).

Femoral head	CTR SHAM (sham-operated controls, *N* = 10)	CTR OVX (ovariectomized controls, *N* = 10)	TTA OVX (ovariectomized, given TTA, *N* = 10)
Cortical volume (Ct.V) (*μ*m^3^)	31.98 ± 2.449	28.26 ± 1.491^aaa^	29.23 ± 2.110^a^
Cortical thickness (Ct.Th) (*μ*m)	449.4 ± 18.80	422.3 ± 21.21^aa^	422.9 ± 16.89^aa^
Trabecular bone volume (BV) (*μ*m^3^)	10.94 ± 1.365	10.69 ± 1.291	11.32 ± 1.465
Total volume (TV) (*μ*m^3^)	20.12 ± 2.062	23.70 ± 2.926^aa^	25.38 ± 2.920^aaa^
Trabecular thickness (Tb.Th) (*μ*m)	155.2 ± 6.893	147.2 ± 3.445^aa^	149.9 ± 3.809
Connectivity density (CD) (1/mm^3^)	44.42 ± 9.160	46.34 ± 7.634	45.50 ± 7.698
Structure model index (SMI) (0.0–3.0)	0.6509 ± 0.2211	0.9811 ± 0.1391^aa^	1.037 ± 0.2294^aaa^
Trabecular bone volume fraction (BV/TV)	0.5436 ± 0.04273	0.4511 ± 0.02421^aaa^	0.4480 ± 0.03645^aaa^

^a, aa, aaa^ *P* < 0.05, *P* < 0.01, *P* < 0.001 when compared to CTR SHAM group.

**Table 6:** Mean values ± SD of bone architecture parameters in the femoral metaphysis, determined by means of *μ*CT scanning analyses in sham controls, ovariectomized controls, and ovariectomized rats fed TTA for four months (Study II).

Femoral metaphysis	CTR SHAM (sham-operated controls, *N* = 10)	CTR OVX (ovariectomized controls, *N* = 10)	TTA OVX (ovariectomized, given TTA, *N* = 10)
Cortical volume (Ct.V) (*μ*m^3^)	28.77 ± 1.773	27.13 ± 1.551^a^	28.39 ± 2.000
Cortical thickness (Ct.Th) (*μ*m)	735.5 ± 22.90	694.4 ± 41.84^a^	693.8 ± 28.02^aa^
Trabecular bone volume (BV) (*μ*m^3^)	6.554 ± 0.8317	5.147 ± 0.5373^aa^	5.300 ± 2.20^a^
Total volume (EV) (*μ*m^3^)	21.85 ± 1.640	25.23 ± 1.734^aaa^	25.88 ± 2.777^aaa^
Trabecular thickness (Tb.Th) (*μ*m)	148.3 ± 5.638	146.7 ± 6.034	147.5 ± 6.927
Connectivity density (CD) (1/mm^3^)	14.20 ± 5.740	10.91 ± 2.118	10.97 ± 3.978
Structure model index (SMI) (0.0–3.0)	0.6682 ± 0.2296	1.596 ± 0.2397^aaa^	1.578 ± 0.1939^aaa^
Trabecular bone volume fraction (BV/TV)	0.2991 ± 0.03208	0.2056 ± 0.02008^aaa^	0.2030 ± 0.02983^aaa^

^a,  aa,  aaa^ *P* < 0.05, *P* < 0.01, *P* < 0.001  when compared to CTR SHAM group.

**Table 7:** Biomechanical properties of the femoral neck and shaft in mean values ± SD in sham controls, ovariectomized controls, and ovariectomized rats fed TTA for four months (Study II).

	CTR SHAM (sham-operated controls) (*N* = 10)	CTR OVX (ovariectomized controls) (*N* = 10)	TTA OVX (ovariectomized, given TTA) (*N* = 10)
Ultimate bending moment (Nm)			
Femoral neck	63.0 ± 8.9	52.1 ± 8.9^a^	49.6 ± 13.3^a^
Femoral shaft	81.7 ± 6.3	76.8 ± 7.6	75.8 ± 7.6
Energy absorption (J × 10^−2^)			
Femoral neck	13.5 ± 2.7	9.94 ± 2.6^a^	11.9 ± 4.7
Femoral shaft	15.3 ± 2.3	13.3 ± 2.8	15.1 ± 3.0
Bending stiffness (Nm/° × 10^−2^)			
Femoral neck	370 ± 58	317 ± 53	290 ± 105
Femoral shaft	574 ± 61	582 ± 58	510 ± 92

^a^ *P* < 0.05  when compared to CTR SHAM group.
